# Vortex formation time in female athletes

**DOI:** 10.1007/s10554-023-02995-8

**Published:** 2023-11-27

**Authors:** Stefan Cirovic, Andreas Malmgren, Rayane Kurdie, Dejan Bilal, Magnus Dencker, Petri Gudmundsson

**Affiliations:** 1 Biomedical Sciences, Faculty of Health and Society, Malmö University, Malmö, 205 06 Sweden; 2 Clinical Physiology and Nuclear Medicine, Skåne University Hospital, Malmö, 205 06 Sweden

**Keywords:** New screening methods, Vortex formation time, Doppler analysis, Female participants

## Abstract

Regular, vigorous physical activity can have a significant impact on cardiac function, leading to cardiac morphological alterations that may be challenging to distinguish from pathological changes. Therefore, new screening methods are needed to accurately differentiate between adaptive changes and pathological alterations in athletes. Vortex formation time (VFT) is an emerging method that shows potential in this regard, as it involves the formation of a rotating vortex ring in the left ventricle during the early filling phase of diastole. In this study, we investigated the difference in VFT between two groups of women: professional handball players and healthy middle-aged female athletes, along with their corresponding control groups. By using echocardiography-Doppler analysis of the heart, VFT was calculated based on the left ventricular ejection fraction, the ratio between the end-diastolic volume and the diameter of the mitral annulus, and the ratio of the atrial contraction volume to the total inflow via the mitral valve. The study reveals a significant increase in VFT in both professional handball players and middle-aged female athletes compared to their respective control groups. Moreover, statistically significant differences between handball players and middle-aged female athletes were observed, indicating that the level of physical activity may affect the VFT. These results suggest that VFT could be a promising screening tool for identifying cardiac adaptations due to long-term vigorous training, potentially enabling more accurate diagnoses of cardiac morphological alterations in athletes.

**Graphical abstract:**

Representation of the graphical abstract of the conducted research 
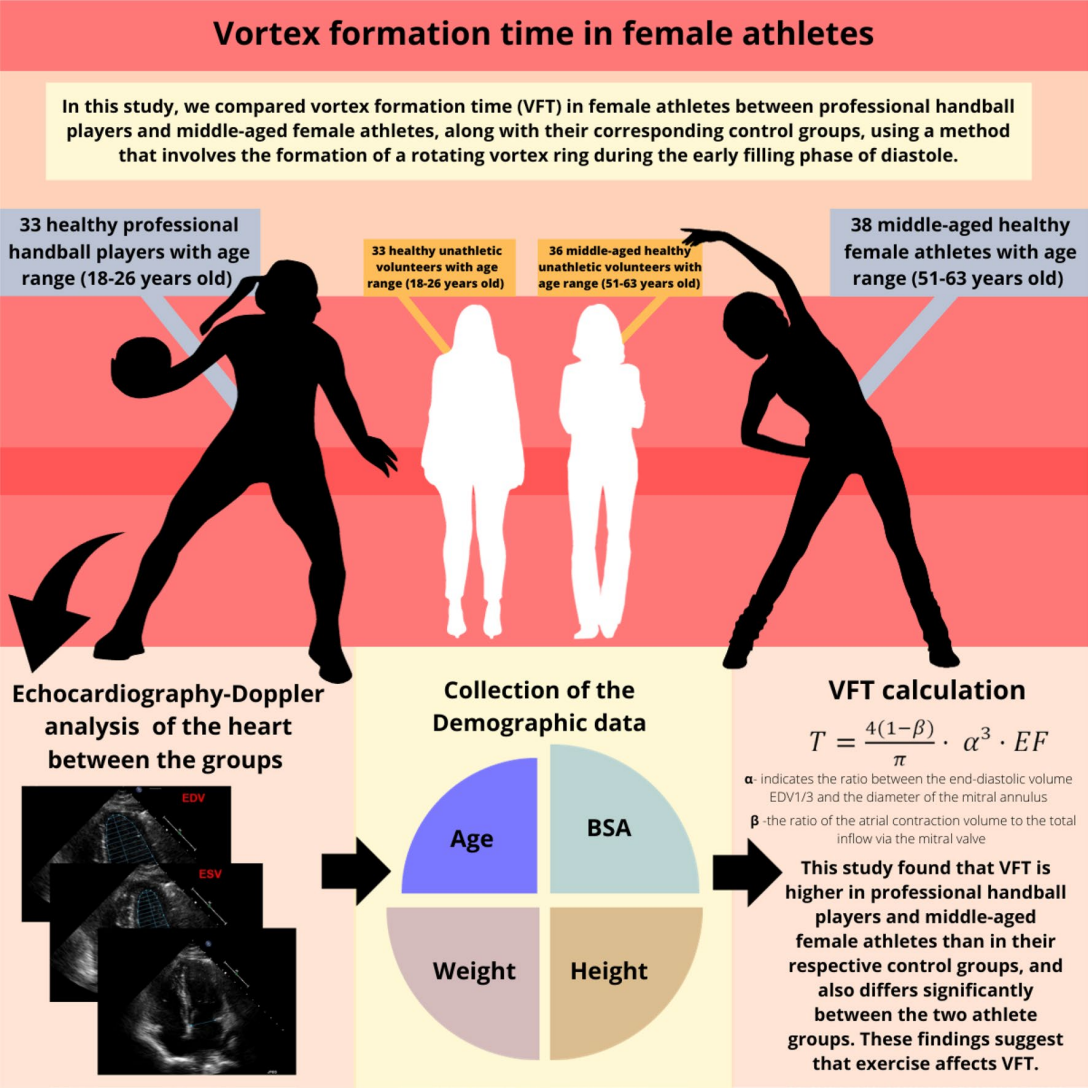

## Introduction

Function of the myocardium can be described by the relaxation ability of the left ventricle. During physical activity, the body undergoes various physiological changes which are a consequence of the excessive need for the energy production [1–3]. These changes include the change of the function and structure of the heart which plays a crucial role in energy distribution over the body [4, 5]. Engaging in physical activity is widely acknowledged as highly beneficial for treatment of various diseases [6, 7]. Physiological phenomenon characterized by structural and functional adaptations to regular physical exercise is called ‘‘athlete’s heart’’ [7]. Recent studies have shown that long impulsive training can lead to left ventricular dilatation and hypertrophy, as well as an increase in left ventricular mass. Such changes can be identified with the echocardiography-Doppler analysis which can examine the ability of filling capacity of the heart [5, 8]. Indeed, knowing the filling capacity can be crucial for the athletes allowing their cardiac function to be assessed and used for the further development in their body physique and sports. One of the measurements that can be obtained through heart echocardiography is vortex formation time (VFT), which explains the process of vortex formation within the left ventricle (LV) and contributes to a better understanding of diastolic left ventricular function [9–11]. According to prior research, the morphological structure and physiological function of the heart vary among athletes who engage in different physical activities. One example is the difference in the hearts between strength-trained and endurance-trained individuals. During the performance of the physical exercise body undergoes many different morphological and chemical changes which can affect heart and its function [2, 3]. Previous studies suggested that “endurance-trained” heart would mainly lead to eccentric LV hypertrophy while a “strength-trained” heart would lead predominantly to concentric LV hypertrophy. However, recent studies suggest that there are no universal upper normal limits for LV dimensions correlated to different sporting disciplines. The concept of sport-specific standard values could serve as a method for identifying athletes whose cardiac dimensions, in relation to their gender and sport specialty, deviate from the norm. Therefore, while a certain degree of association may exist between training routines and cardiac adaptation patterns, this relationship is intricate and subject to variation, influenced by both the sport type and individual athlete [12]. Nevertheless, studies suggest that endurance exercise can induce sustained muscular strain, which, particularly in athletes, may contribute to increase of cardiac output and its consequent impact on blood pressure. Cardiac output is increased mainly from a surge in heart rate and stroke volume [13]. During the increase of the physical activity the heart increases its stroke volume, up to the maximal heart rate of 150 bpm. Beyond the maximal heart rate value, the stroke volume declines due to the shortened diastolic filling time [14]. However, the cardiac output continues to increase due to the combination of a higher heart rate and an improved ejection fraction. Moreover, the expansion of blood vessels within the active muscles, brought about by a reduction in peripheral vascular resistance, significantly contributes to the overall increase in cardiac output. Change in pressure can cause left ventricular dilation and thickening which leads to the increase of the left ventricular diameter with a proportional increase in ventricular thickness [5, 8, 15–17].

### Cardiac properties and health status of an athlete

The adaptation of the heart is contingent on the specific nature of the exercise performed. Participating in elite-level endurance exercises like swimming, cycling, or running increases cardiac output, which, in turn, leads to increased blood pressure due to long-term muscular stress [1, 2]. As a result of the volume load, there is an increase in the left ventricular diameter and a corresponding increase in ventricular thickness (eccentric hypertrophy) [5]. This adaptation is the heart’s way of responding to the demands placed upon it during sustained aerobic exercise. Structural changes are essential for enhancing cardiac output, enabling the heart to effectively pump a larger volume of blood per beat. During endurance training, the body requires increased oxygen and nutrient delivery to active muscles, leading to a higher cardiac output. Eccentric hypertrophy helps to meet this demand by optimizing the heart’s ability to pump larger quantities of blood with each contraction. This adaptation is beneficial for athletes engaged in activities like long-distance running, swimming, or cycling [14]. Pressure overload and the subsequent increase in peripheral resistances that occur during high-intensity strength training, particularly in competitive athletes, are relatively brief physiological occurrences. Following this transient period of increased pressure, a compensatory vasodilation response ensues, effectively returning the athlete’s blood pressure to normal levels or, in some cases, even lowering it. This differentiation is essential, as it signifies that the brief pressure surge associated with strength training does not result in a lasting blood pressure increase, in contrast to conditions such as arterial hypertension or left ventricular outflow tract stenosis, where blood pressure remains chronically elevated [18]. Despite this thickening, the volume of blood that the LV can hold at the end of diastole remains unchanged. Considering the load on the heart recent studies suggest that long term exercise does not change the geometry of the heart in athletes and people who undergo rigorous training exercises [12, 19]. Consequently, it’s evident that these athletes encounter both volume load and pressure stress. A meta-analysis highlights that individuals participating in a combination of endurance and strength training possess increased left ventricular wall thickness and capacity in comparison to those exclusively engaged in either endurance or strength training [20]. In relation to left ventricular hypertrophy, both endurance and strength training contribute to a greater relaxation of the LV. It has also been demonstrated that an increase in left ventricular relaxation contributes to enhanced diastolic function in elite athletes [21]. There is an increase in cardiac mass and left ventricular hypertrophy in systemic hypertension. Alternatively, hypertrophy is related to ventricular stiffness and decreased relaxation in the LV [1, 21]. Since ventricular stiffness affects the filling of the LV and thus the formation of vortex in the LV, VFT is a potential variable to differentiate between pathological changes that occur in the LV during systemic hypertension and prolonged intense exercise [5].

### Vortex formation and relation to the diseases

Previous studies have shown that diastolic filling dynamics of the LV play a crucial role in determining cardiac health. Early diastolic filling of the LV, or the E wave, is characterized by the formation of a vortex ring, a rotating fluid mass observed in both in vivo and in vitro studies [9, 22, 23]. Fluid transport by vortex ring formation has been shown to be more efficient than by a steady, straight jet of fluid, according to extensive in vitro experiments studying this process. In addition, it was recently found that vortex rings’ maximum growth is constrained by energetic considerations [24]. These findings point to the potential for optimization of vortex ring formation in fluid transport processes occurring in nature, which is particularly important for biological systems that rely on such processes for their survival. Vortex formation can, in theory, dictate the optimal kinematics of any biological fluid transport system, including the human heart, according to a study that used in vivo and in vitro data to back up this claim [25].

### Vortex formation time and ejection fraction

Ejection fraction (EF) is measured when examining left ventricular systolic function. EF is expressed as a percentage and is defined as the proportion of the end-diastolic volume that is ejected during systole. According to the guidelines, the recommended method for calculating EF is Simpson’s biplane method [26–28].

1 $$EF\% = \frac{{End~Diastolic~Volume~\left( {EDV} \right) - End~Systolic~Volume~\left( {ESV} \right)}}{{EDV}}~ \times 100$$

VFT is a dimensionless index used to measure vortex formation. As the index is dimensionless, it has the potential to be a useful measurement [29]. In previous studies, VFT was determined in vitro using a piston/cylinder design in a water tank based on average flow speed U_t_, liquid duration (t), and pipe diameter (D) [24]. According to the study such correlation can be described with the Eq. 2.

2 $$T = \frac{{U_{t} \cdot t}}{D} = \frac{L}{D}$$

Thus, the equation can be expressed as a ratio of the length (L) to the diameter of the flow (D) [24]. According to the studies the maximal VFT is T ≈ 4 which is the most efficient and optimal transport of liquid [24]. By incorporating factors that impact blood flow from the left atrium (LA) to the LV, the equation may be altered to estimate the left ventricle’s vortex ring. Since mitral valve flow is linked to LV function, an EF-based index has been established according to the Eq. 3 and Eq. 4.

3 $$T = \frac{{4(1 - \beta )}}{\pi } \cdot \alpha ^{3} \cdot EF$$

4 $$~\alpha = \frac{{EDV^{{1/3}} }}{{\bar{D}}}~$$

where indicates the ratio between the end-diastolic volume EDV^1/3^ and the diameter of the mitral annulus. Variable β represents the ratio of the atrial contraction volume to the total inflow via the mitral valve [24]. The range of VFT during normal left ventricular function is 3.3 to 5.5, as determined by previous in vitro vortex formation studies and observations of optimal VFT. Mitral valve stenosis and pathological conditions that impact mitral annulus diameter can result in an increase in VFT, whereas a reduction in EF can lead to a decrease in VFT by approximately 1.5 to 2.5. [24].

For the calculation of variable β needed for the further calculation of VFT, the variables from the Eq. 5 were used.

5 $$~~~\beta = ~\frac{{VTI_{A} }}{{VTI_{E} + VTI_{A} }}~$$

Velocity time integral A (VTI_A_) and Velocity time integral E (VTI_E_) were measured in order to determine, which is the ratio of VTI_A_ to the total transmittal input (i.e., VTI_E_ + VTI_A_). The determination of the inflow through the mitral valve is performed by using the pulsed doppler measurement method. The E and A waves were found while monitoring mitral inflow. The VTI is expressed in centimeters and is the inflow’s average velocity (cm/s) multiplied by the ejection time [29].

### Mitral inflow and diastolic function

During the process of systole, blood is pumped through the aortic valve from the left ventricle. This results in the decrease of pressure in LV and the closure of aortic valve [30]. It comprises of four phases: Isovolumic relaxation time, fast filling, diastasis, and atrial contraction. After aortic valve closure, Isovolumic relaxation time (IVRT) occurs. This is followed by a rapid drop in left ventricular pressure and myocardial relaxation. The ending of IVRT appears when the pressure of the LA drops below the pressure in LV, causing the mitral valve to open [31]. During the rapid filling phase of the cardiac cycle, the flow of blood from the LA to the LV is induced by a pressure differential between the two chambers, with higher pressure in the LA causing blood to flow into the lower pressure in LV. During diastasis, the pressure difference between the left atrium and left ventricle is gradually equalized as blood flows into the ventricle, resulting in a period of decreased blood flow velocity in the absence of significant pressure gradients. Atrial contractions fill the LV as left atrial pressure surpasses LV pressure. Atrial contraction contributes 20% to healthy left ventricular filling [31, 32]. Using a pulsed wave doppler placed between the mitral valve tips in the apical four-chamber projection and measuring mitral inflow, the diastolic function of the LV can be evaluated. With pulsed wave doppler, the maximum speed during rapid filling (E) and atrial contraction (A) can be measured to characterize diastolic blood flow across the mitral valve [31].

## Materials and methods

Echocardiography of the heart and data gathering were performed on 140 female volunteers at the Department of Clinical Physiology and Nuclear Medicine, Skåne University Hospital, Malmö. The study involved 140 female volunteers, including 33 healthy professional handball players who regularly perform dynamic and static training. A corresponding control group of 33 healthy female volunteers was also included, consisting of untrained individuals of the same age range (18–26 years old). Additionally, there were 38 middle-aged healthy female athletes who regularly train, along with a corresponding group of 36 healthy female volunteers all of whom were aged between 51 and 63 years old. A biomedical scientist with the appropriate credentials conducted a transthoracic examination. The iE33 ultrasound system manufactured by Philips Medical Systems, Andover, MA, USA with the s3 transducer was used to perform two-dimensional echocardiography and Doppler imaging. To obtain the necessary cardiac images, a four-chamber view was acquired, which required the participants to be shirtless during the evaluation. The optimal positioning for acquiring this view was in a reclined position on the left side, with the left arm placed beneath the participant’s head. During image analysis, a unipolar electrocardiogram (ECG) was incorporated into the echocardiography machine to monitor the cardiac cycle. Time gain compensation (TGC) was utilized to enhance the image quality.

### Cardiovascular measurements

**Fig. 1:**
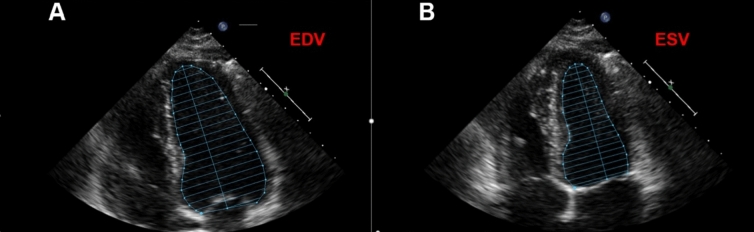
Measurements of EDV and ESV used in Simpson’s biplane method for the calculation of EF

The measurements of Ejection Fraction (EF), End diastolic volume (EDV), End systolic volume (ESV), *annulus mitralis* diameter and Velocity time integrals were performed in a software Philips IntelliSpace Cardiovascular 5.2 made by Koninklijke Philips N.V. Philips Medical Systems, the Nederlands. The average value determination of EDV and ESV was performed in apical four-chamber projection and the apical two-chamber projection. Determination of EF was calculated with Simpson’s biplane method by manually aligning the endocardium at end-diastole and end-systole in apical four-chamber projection as shown in Fig. 1.

**Fig. 2:**
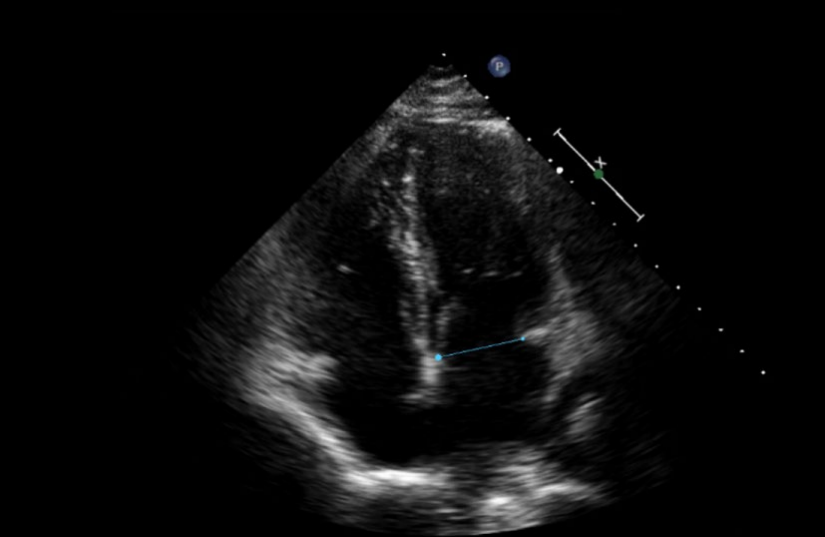
Measurements of mitral annulus during early diastole

The diameter of the mitral annulus was measured during early diastole, while the mitral valve was open. In the four-chamber projection, a line was manually drawn between the anterior and posterior mitral ends in order to obtain the result of the measurement as shown in Fig. 2.

**Fig. 3:**
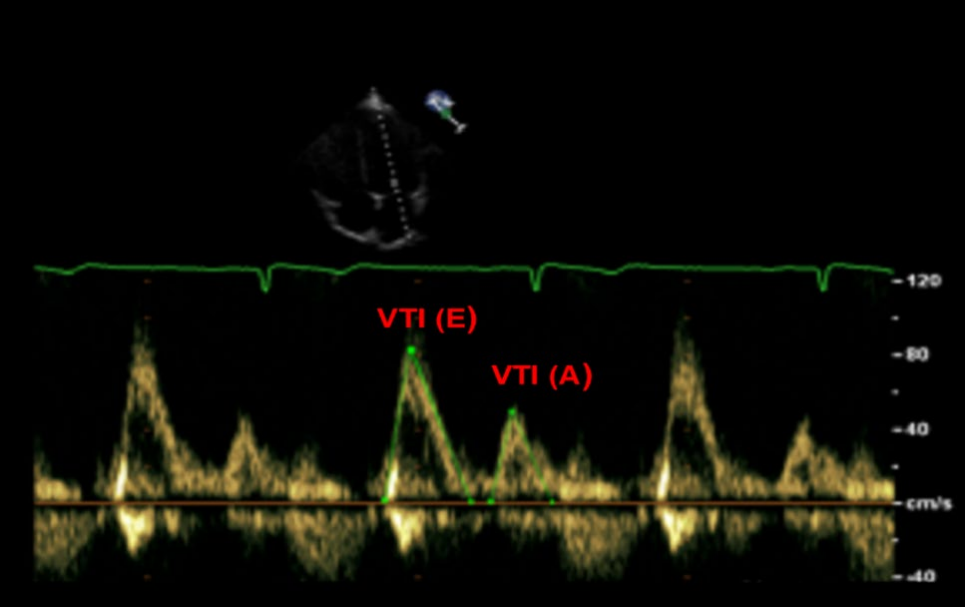
VTI measurements taken during early diastole (E) and atrial contractions (A)

 The mitral valve inflow was recorded using pulsed wave doppler. The sample volume was placed between the mitral valve’s tip in an apical four-chamber projection for the duration of the measurement. During the measurement of mitral input, E and A waves were recorded. The E wave VTI (VTI (E)) and A wave VTI (VTI(A)) were determined by plotting the area of the spectral curve which can be seen in Fig. 3.

The VFT is obtained by applying Eq. 3, which consists of the mean value of the EDV (A), the *annulus mitralis* diameter and β.

### Statistical analysis

The Statistical analysis were performed with IBM, SPSS Statistics, version 27, Chicago, IL, USA. A student t-test with a 95% confidence interval was performed in order to determine whether there was a statistically significant difference between the groups on all variables. Wilks’ Lambda test (MANOVA) was used to assess the existence of significant statistical variations between the groups.

## Results

Based on the implementation of the practical methodology of analysis described in methods section *vide supra* the gathered data was compared and analyzed with regard to the groups of interest and the control groups to investigate possible correlation.

Results presented in Table 1 provide the mean value, standard deviation, and P-value for the t-test of echocardiographic measures. There are notable differences in parameters between the handball control group and the professional handball players in terms of heart rate, EDV, ESV and VFT. The VFT of the professional handball players was substantially greater than that of the corresponding control group. The mitral annulus diameter did not reveal any significant differences and the obtained P-value suggests that there is no significant relationship between the results. However, there was a significant difference of heart rate values between the groups with a P value lower than 0.001.

**Table 1:** Comparison between the handball control group and professional handball players

Parameter	Groups		
	Handball control group (Mean ± SD) n = 33	Professional handball players (Mean ± SD) n = 33	P-value
Heart rate (beats/min)	70.00 ± 13.00	56.00 ± 8.00	< 0.001
EF (%)	61.5 ± 3.60	58.3 ± 3.30	< 0.001
EDV (ml)	92.7 ± 12.90	129.00 ± 18.00	< 0.001
ESV (ml)	35.8 ± 5.40	54.00 ± 8.10	< 0.001
Diameter of mitral annulus (cm)	3.14 ± 0.24	3.10 ± 0.33	> 0.1
VFT	1.85 ± 0.48	2.63 ± 0.71	< 0.001

*EF *Ejection fraction, *EDV *End diastolic volume, *ESV *End systolic volume, *VFT *Vortex formation time

The demographic information for the handball control group and the professional handball players is presented in Table 2. All parameters are presented using the format mean value standard deviation (SD). Group differences in age, body surface area (BSA), weight, and height were statistically significant. What is notable is that weight and BSA of the professional handball players is slightly higher.

**Table 2:** Demographic statistics on handball players and a handball control group

Parameter	Groups		
	Handball control group (Mean ± SD) n = 33	Professional handball players (Mean ± SD) n = 33	P-value
BSA (m^2^)	1.7 ± 0.11	1.9 ± 0.13	< 0.001
Weight (kg)	64 ± 8.60	73 ± 7.90	< 0.001
Height (cm)	171 ± 5.50	175 ± 6.90	< 0.05
Age (years)	23 ± 2	20 ± 2	< 0.001

*BSA *Body surface area

The Table 3 shows the comparison of various parameters between middle-aged female athletes’ group with their corresponding group. The mean and standard deviation of each parameter are shown for both groups, along with the P-value indicating the statistical significance of the difference between the two groups. The results indicate that there were statistically significant differences between the two groups in heart rate, EDV, ESV, and diameter of mitral annulus, but no significant difference in EF and VFT.

**Table 3:** Comparison between the middle-aged control group and middle-aged female athletes

Parameter	Groups		
	Middle-aged control group (Mean ± SD) n = 36	Middle-aged female athletes (Mean ± SD) n = 38	P-value
Heart rate (beats/min)	66.0 ± 9.00	55.0 ± 7.00	> 0.001
EF (%)	67.2 ± 4.90	67.2 ± 4.02	> 0.1
EDV (ml)	89.1 ± 15.20	108.3 ± 16.50	< 0.001
ESV (ml)	29.1 ± 6.5	35.7 ± 8.10	< 0.001
Diameter of mitral annulus (cm)	2.9 ± 2.9	3.0 ± 3.20	< 0.05
VFT	1.99 ± 0.58	2.2 ± 0.74	> 0.1

*EF *Ejection fraction, *EDV *End diastolic volume, *ESV *End systolic volume, *VFT *Vortex formation time

Table 4 presents the demographic information for the control group and the middle-aged female athletes. Gathered results indicate that there were no significant differences between the groups. BSA and weight are statistically significant based on the P-value results whereas height indicates low significance between the groups.

**Table 4:** Demographic statistics on middle-aged female athletes and a middle-aged control group

Parameter	Groups		
	Middle-aged control group (Mean ± SD) n = 36	Middle-aged female athletes (Mean ± SD) n = 38	P-value
BSA (m^2^)	1.8 ± 0.10	1.7 ± 0.10	> 0.01
Weight (kg)	68 ± 9.80	61 ± 7.20	< 0.001
Height (cm)	166 ± 5.20	165 ± 6.90	> 0.1
Age (years)	58 ± 3	57 ± 3	> 0.1

*BSA *Body surface area

Table 5 presents the findings of the comparison between the 33 professional handball players and the 38 middle-aged female athletes. Results suggest there is a significant difference between the parameters, with the exception of the diameter of the mitral annulus and VFT, where both parameters exhibit a lower correlation. The analysis reveals that the heart rates of the two physically active groups exhibit no statistically significant difference.

**Table 5:** Comparison between the professional handball players and middle-aged female athletes

Parameter	Groups		
	Professional handball players (Mean ± SD) n = 33	Middle-aged female athletes (Mean ± SD) n = 38	P-value
Heart rate (beats/min)	56.00 ± 8.00	55.0 ± 7.00	> 0.01
EF (%)	58.3 ± 3.30	67.2 ± 4.02	< 0.001
EDV (ml)	129.0 ± 18.00	108.3 ± 16.5	< 0.001
ESV (ml)	54.0 ± 8.10	35.7 ± 8.10	< 0.001
Diameter of mitral annulus (cm)	3.10 ± 0.33	3.0 ± 3.20	> 0.1
VFT	2.63 ± 0.71	2.2 ± 0.74	< 0.05

*EF *Ejection fraction, *EDV *End diastolic volume, *ESV *End systolic volume, *VFT *Vortex formation time

Table 6 presents the demographic information for the professional handball players and the middle-aged female athletes. The P-value indicates the statistical significance of the differences between the two groups for each parameter, with values less than 0.05 indicating a significant difference. Overall, the professional handball players had larger BSA, weight, and height, and were younger than the middle-aged female athletes.

**Table 6:** Demographic comparison between professional handball players and middle-aged female athletes

Parameter	Groups		
	Professional handball players (Mean ± SD) n = 33	Middle-aged female athletes (Mean ± SD) n = 38	P-value
BSA (m^2^)	1.9 ± 0.13	1.7 ± 0.10	< 0.001
Weight (kg)	73 ± 7.90	61 ± 7.20	< 0.001
Height (cm)	175 ± 6.90	165 ± 6.90	< 0.001
Age (years)	20 ± 2	57 ± 3	< 0.001

*BSA *Body surface area

Comparison between the handball control group and middle-aged control group is presented in Table 7. The results indicate that there are statistically significant differences between the two groups for most parameters, including heart rate, EF, ESV, diameter of the mitral annulus, and EDV.

**Table 7:** Comparison between handball control group and middle-aged control group

Parameter	Groups		
	Handball control group (Mean ± SD) n = 33	Middle-aged control group (Mean ± SD) n = 36	P-value
Heart rate (beats/min)	70.00 ± 13.00	66.0 ± 9.00	< 0.001
EF (%)	61.4 ± 3.56	67.2 ± 4.02	< 0.001
EDV (ml)	92.6 ± 18.00	89.1 ± 15.2	> 0.01
ESV (ml)	29.1 ± 5.50	35.7 ± 8.10	< 0.001
Diameter of mitral annulus (cm)	3.10 ± 0.33	2.9 ± 0.29	< 0.001
VFT	1.84 ± 0.48	1.9 ± 0.56	> 0.1

*EF *Ejection fraction, *EDV *End diastolic volume, *ESV *End systolic volume, *VFT *Vortex formation time

Demographic comparison between two control groups is shown in Table 8. Obtained results show low correlation between the parameters. Results obtained between the heights of each groups participants with the P-value of < 0.001 indicate greater correlation than the rest of the parameters found in Table 8.

**Table 8:** Demographic comparison between handball control group and middle-aged control group

Parameter	Groups		
	Handball control group (Mean ± SD) n = 33	Middle-aged control group (Mean ± SD) n = 36	P value
BSA (m^2^)	1.7 ± 0.11	1.8 ± 0.13	> 0.1
Weight (kg)	64.4 ± 8.62	68.3 ± 9.75	> 0.05
Height (cm)	170.8 ± 5.50	165.8 ± 0.87	< 0.001
Age (years)	23 ± 2	58 ± 3	< 0.001

*BSA *Body surface area

Table 9 presents the results of a Wilks’ Lambda test for the comparison of EF, EDV, and VFT among the groups.

**Table 9:** Multivariate Wilks’ Lambda test between EF, EDV and VFT

	Value	F	Hypothesis df	Error df	Sig. (P)	Partial Eta Squared (η^2^)
Professional handball players group with the corresponding control group	0.297	49.018^b^	3.000	62.000	< 0.001	0.703
Middle-aged female athletes with the corresponding control group	0.703	9.856^b^	3.000	70.000	< 0.001	0.297

The handball group showed a smaller Wilks’ Lambda value of 0.297, while the middle-aged group showed a larger value of 0.703, indicating greater differences between the groups. The F-value indicates the degree of difference between the groups relative to the variation within the groups. A larger F-value suggests a greater gap between the groups. In this instance, both the handball group (F = 49.018) and the middle-aged group (F = 9.856) differed significantly from their respective control groups, as demonstrated by the extremely low P-values (0.001). The P-value implies the level of statistical significance, with a value of < 0.001 indicating a very low probability that the differences observed between the groups were due to chance. Effect size (η2) is a measure of how much the differences between groups can explain the overall variation in the data. In this case, the handball group had a large effect size (η2 = 0.703), which means that a significant amount of the variation in the data can be explained by the differences between the handball and control groups. On the other hand, the middle-aged group had a smaller effect size (η2 = 0.297), suggesting that there was less variation in the data that could be attributed to differences between the middle-aged and control groups.

## Discussion

The decision to include a middle-aged group within our study stemmed from our intent to offer a more encompassing perspective across various age cohorts, thereby enhancing the applicability and relevance of our findings. Different diastolic function and vortex dynamics across various age groups is to be expected since the cardiovascular system undergoes age-related transformations, leading to distinct diastolic performance and vortex patterns. Having middle-aged participants involved in the study we aimed to explore the impact of age on vortex dynamics and diastolic function. Introduction of age diversity provides us with a more comprehensive understanding of how diastolic function and vortex parameters evolve over time. This inclusive approach not only extends the reach of our findings to a more diverse demographic but also sheds light on the intricate interplay between age-related cardiac adaptations and the phenomenon of vortex formation.

VFT of professional handball players was substantially greater than that of the healthy control group, according to this research. This indicates that endurance-trained people had enhanced diastolic function and a greater capacity to relax during diastole, compared to the healthy control group. The findings of this study are consistent with those of prior similar research, indicating that VFT may be a helpful measure for differentiating between pathological and sporting cardiac alterations [11, 33]. VFT in middle-aged female athletes when compared to the corresponding group did not show significant changes despite regular physical activities. Comparison between professional handball players and middle-aged female athletes suggests that high-level professional sporting activities have a higher impact on VFT than consistent mild sporting activities performed by middle-aged female athletes. The gap difference in VFT between these two groups is noticeable with a P-value < 0.05, which indicates relatively strong evidence against the null hypothesis.

In addition to this, the demographic comparison between professional handball players and middle-aged female athletes indicates that professional handball players, who engage in more extensive physical activities, have a more advanced body structure compared to middle-aged female athletes. Such results can be observed in Table 6, where height, weight, and BSA are significantly higher in the professional handball players than in middle-aged female athletes. It can be inferred that the professional handball players displayed significant differences between the parameters, as indicated by statistical analysis. These differences were not consistent with a normal distribution. The athletic middle-aged women showed no significant variation in VFT, and the values did not follow a normal distribution. However, the comparison between the handball control group and the middle-age control group could not be correlated since they are not athletes or physically active individuals.

### VFT between the groups

When compared to the normal range of VFT, the mean value was significantly lower than the usual range in both professional handball players, middle-aged female athletes as well as in the control groups. According to the literature, the normal range for VFT during normal left ventricular function is 3.3 to 5.5 [21]. When comparing the results of the professional handball players and middle-aged female athletes the mean for VFT is 2.63 ± 0.71 respective 2.2 ± 0.74 which is seen as a normal result below the norm of VFT 3.3. Similar results were obtained in both control groups where handball control groups had significantly lower VFT 1.84 ± 0.48 as well as the middle-aged control group VFT 1.90 ± 0.56. Previous research indicates that the VFT lower than the norm does not exclude the creation of a vortex ring [22]. Consequently, the obtained findings are entirely interpretable in terms of VFT according to the sources. All patients in this research had a comprehensive echocardiographic assessment, and all showed normal heart function. Prior to that finding, no medical issues had been reported, and none of the participants had a chronic disease. According to the findings of this research, younger female handball players had a considerably higher VFT than the healthy handball control group as well as the middle-aged female athletes’ group. Previous studies suggest that the adaptability and morphological change of the heart in professional athletes is closely correlated with the sort of training they do which can influence VFT [33, 34]. In current study, VFT was investigated in professional handball players who perform both dynamic and static training. The outcome of the results demonstrated an increase in VFT among handball players. What is notable is that the heart rate in trained individuals, both professional handball players and middle-aged trained individuals was lower when compared to their corresponding control group. The results of the VFT indicate that trained individuals obtained higher VFT values in the female athletes compared to their respective control groups. A previous study involving a group of male athletes and control groups produced similar results, showing a significant increase in VFT among elite athletes. [33]. Since similar findings were observed in the current study, it is plausible to suggest that VFT could serve as a valuable performance indicator in elite athletes, irrespective of gender. This parameter may reflect the physiological changes associated with training. However, further research is necessary to validate these findings and ascertain the clinical significance of VFT in both athletes and non-athletes.

Compared to the healthy control group, the handball players in this research showed considerably higher EDV and ESV values, indicating an eccentric remodeling of the LV. Middle-aged female athletes who participated in the study underwent more modest physical activity suitable for their age. Findings in this group when compared to the relevant control group suggest that EDV and ESV are significantly higher to the control group but not higher than the results obtained in group of the professional handball players. The result in middle-aged female athletes also indicates an eccentric remodeling of the LV but not to that extent as in the group of professional handball players. Higher EDV and ESV in both groups of professional handball players and middle-aged female athletes may have also impacted the VFT, resulting in a greater VFT in handball players compared to the healthy control group. Previous scientific research shows similar results where VFT was measured in a group of older men who were involved in endurance and strength training which consisted of jogging, swimming, and cycling [33]. According to the research, the cardiac adaptation that happens during long-term rigorous training may have led to a more efficient filling capacity in the LV, resulting in a greater VFT in the professional athletes compared to the control group [33].

The multivariate Wilks’ Lambda test resulted into two very different F-values, indicating a substantial variation between the two groups. Similarly, the test conducted on the middle-aged control group revealed a significant difference, with a higher Wilks’ Lambda value and lower F-value. The low P-value (< 0.001) indicates that the differences between the groups are unlikely. Thus, the analysis establishes significant distinctions in EF, EDV, and VFT between the handball and control groups, as well as between the middle-aged and control groups.

### Calculations of VFT

In recent research VFT was calculated by implementing various calculation methods [35, 36]. In a current study, the VFT time was calculated by using the Eq. 3. The mitral annulus diameter was measured at the beginning of early diastole when the mitral valve was completely open. Such approach may have impacted the calculated results and made it difficult to compare them to those of previous VFT investigations.

### Limitation with data gathering and VFT

There was also a considerable variation in age distribution across the groups, which may have impacted the reported findings. Left ventricular wall thickness and left ventricular mass were not included in this study since they were not the subject of the research. However, these variables may have affected the study’s findings. Current study does not include data such as VTI as well as the additional dimensional/volume data and more detailed data regarding left ventricular diastolic function. In the present research, *mitralis* dimensions were measured at the tip of the mitral leaflets, resulting in lower *mitralis* dimensions than if the measurements were done in the annulus region. According to the study where the measurements were done on mitral valve influenced by stenosis, the smaller *mitralis* dimensions resulted into higher VFT [37]. Having the measurements done in annulus region could potentially result into lower values of VFT. However, present measurements were conducted in accordance with the professional standard analysis performed on medical patients, and therefore the results are regarded as the most reliable. In addition, the number of participants in the research was rather modest. Therefore, it is necessary to conduct more research with a greater number of participants and a comparable age distribution across the groups. Additionally, it would be advantageous to standardize when during diastole and in which projection to assess the mitral annulus diameter. This is done to eliminate methodological disparities and make findings more comparable.

Study predominantly focuses on the link between VFT and diastolic function. However, it is crucial to underscore the significance of geometrical and kinetic parameters in the creation of vortices. Geometrical parameters concern the structural attributes of the left ventricle and the mitral valve apparatus, which can have a profound impact on vortex dynamics. Similarly, kinetic parameters, relating to blood flow within the left ventricle, influence the direction, velocity, and turbulence of blood flow, thereby affecting vortex formation. Previous study showed that the vortex ring at end-diastole was closely correlated to LV end-diastolic volume where the size of the LV was considered to investigate vortex kinetics [38]. Therefore, the size, shape and kinetics can be the subject to the future studies regarding VFT. Current study focuses mainly on comparing athletes with their control group to find the indicators for understanding VFT withing the groups.

One notable limitation of this study is the absence of volume correction for BSA when comparing parameters between the two distinct study groups since women handball players have larger BSA compared to their control group of women performing modest types of exercises. Difference in BSA can significantly impact left ventricular volumes, making them appear larger, irrespective of the sport practiced and the degree of training. Difference in the volume would substantially affect the calculation of VFT and potentially influence the study results. Further investigations when considering current limitations will contribute to a deeper understanding of the details of cardiac adaptations in athletes.

## Conclusion

Our study uncovers a distinct and statistically significant contrast in VFT among professional handball players, middle-aged female athletes, and their corresponding control groups. Particularly noteworthy is the substantial increase in VFT observed in professional handball players in comparison to the healthy control group. This strongly suggests that the demanding training regimens and physical conditioning undertaken by the handball players have a significant influence on diastolic function. These findings align with earlier research, suggesting the diagnostic potential of VFT in discerning between pathological cardiac adaptations and those induced by exercise.

Additionally, analysis between professional handball players and middle-aged female athletes indicates the impact of the intensity and nature of physical activities on VFT. Furthermore, a demographic examination uncovers notable variations in the physical attributes of the study participants. Professional handball players, who engage in more rigorous physical activities, exhibit significantly greater measurements for height, weight, and BSA compared to middle-aged female athletes. These distinctions hold statistical significance and deviate from a typical distribution pattern. In contrast, middle-aged female athletes engaged in milder physical activities suitable for their age display minimal variation in VFT, with values that also deviate from the normal distribution. The comparison between the handball control group and the middle-aged control group, composed of non-athletic and sedentary individuals, does not yield meaningful correlations. The study highlights the roles of exercise intensity and age in VFT values. These results emphasize the clinical and physiological importance of VFT as a diagnostic tool and the necessity for further investigations.

## Data Availability

Datasets regarding the cardiovascular results of the project participants can be accessed in the patient database at Skåne University Hospital, department of Clinical Physiology and Nuclear Medicine, Malmö Sweden.
